# The RNA binding protein La/SS-B promotes RIG-I-mediated type I and type III IFN responses following Sendai viral infection

**DOI:** 10.1038/s41598-017-15197-9

**Published:** 2017-11-06

**Authors:** Rebecca Mahony, Lindsay Broadbent, Jacen S. Maier-Moore, Ultan F. Power, Caroline A. Jefferies

**Affiliations:** 1 Molecular and Cellular Therapeutics, Royal College of Surgeons in Ireland, 123 St. Stephen’s Green, Dublin 2, Ireland; 2 Centre for Experimental Medicine, Queen’s University Belfast, Medical Biology Centre, 97 Lisburn Road, Belfast, BT9 7BL Northern Ireland; 3 The University of Texas at El Paso College of Health Sciences, Clinical Laboratory Sciences Program, 500 W. University Avenue, El Paso, Texas 79968 USA; 4 Division of Rheumatology, Department of Medicine and Department of Biomedical Sciences, Cedars-Sinai Medical Centre, 8700 Beverly Blvd, Los Angeles, California, 90048 USA

## Abstract

La/SS-B (or La) is a 48 kDa RNA-binding protein and an autoantigen in autoimmune disorders such as systemic lupus erythematosus (SLE) and Sjögren’s syndrome (SS). La involvement in regulating the type I interferon (IFN) response is controversial - acting through both positive and negative regulatory mechanisms; inhibiting the IFN response and enhancing viral growth, or directly inhibiting viral replication. We therefore sought to clarify how La regulates IFN production in response to viral infection. ShRNA knockdown of La in HEK 293 T cells increased Sendai virus infection efficiency, decreased IFN-β, IFN-λ1, and interferon-stimulated chemokine gene expression. In addition, knockdown attenuated CCL-5 and IFN-λ1 secretion. Thus, La has a positive role in enhancing type I and type III IFN production. Mechanistically, we show that La directly binds RIG-I and have mapped this interaction to the CARD domains of RIG-I and the N terminal domain of La. In addition, we showed that this interaction is induced following RIG-I activation and that overexpression of La enhances RIG-I-ligand binding. Together, our results demonstrate a novel role for La in mediating RIG-I-driven responses downstream of viral RNA detection, ultimately leading to enhanced type I and III IFN production and positive regulation of the anti-viral response.

## Introduction

Host viral detection systems rely mostly on recognition of viral nucleic acids by pattern recognition receptors (PRRs) including, RNA and DNA-sensing Toll-like receptors (TLR-3, -7, -8, -9), DNA receptors (DAI, AIM2, IFI16, DDX41) and RIG-I-like receptors (RLRs). RIG-I is an essential type I and type III IFN-inducing receptor required for the detection of negative-sense single stranded RNA viruses such Sendai virus, a member of the *Paramyxoviridae* family, in addition to *Rhabdoviridae* and *Orthomyxoviridae* family members^1–3^. Upon recognition of pathogenic RNA, an ATP-dependent conformational change is triggered in RIG-I exposing the activatory CARD domains. This allows interaction between the second CARD domain of the receptor and the CARD domain of downstream mitochondrial-associated adaptor, IPS-1^4–6^. This interaction leads to assembly and activation of downstream IKK-related kinases TBK-1 and IKK-ε, that subsequently phosphorylate IRF-3 and IRF-7 ^7,8^. This ultimately results in transcriptional induction of both type I and type III IFNs, which in turn leads to robust expression of IFN-stimulated genes (ISGs)^9,10^.

Type I IFNs, including IFN-α, -β, -ω, -κ and -ɛ, act on cells via binding to the IFN-α receptor (IFNαR), comprised of an IFNαR1 and IFNαR2 heterodimer^11,12^. Type I IFN synthesis occurs in virtually all cell types downstream of anti-viral PRR recognition of viral RNA/DNA. Once secreted by the virally-infected cell, type I IFNs bind and activate IFNαR, leading to induction of interferon stimulated genes (ISGs) through activation of JAK1 and Tyk2, followed by phosphorylation of signal-transducing activators of transcription (STAT) proteins STAT1 and STAT2 ^13–16^. ISGs, including RIG-I, TLR-3, OAS1 and OAS2, are expressed following STAT1/STAT2 activation, leading to the inhibition of transcription and translation of viral proteins^17,18^, along with induction and synthesis of MHC class I expression. This makes the cell more susceptible to CD8^+^ cytotoxic T cells^19,20^, activates NK cells which selectively kill virus-infected cells^21,22^, and leads to maturation of DCs^23^ and B cell responses^20,24^.

Functional members of the Type III IFN family, including IFN-λ1 (IL-29), IFN-λ2 (IL-28A) and IFN-λ3 (IL-28B), are induced downstream of TLR-3 and RLR signalling^25,26^ but signal through an independent cell-surface receptor complex, consisting of IL10R2 (also called CRF2–4) and IFN-λR1 (also called IL-28RA)^27,28^. While the type I IFN receptor is ubiquitously expressed, the expression of the IFN-λR1 component of the type III IFN receptor complex appears to be more limited and restricted to cells of epithelial origin, plasmacytoid DCs, macrophages, monocyte-derived DCs and intra-hepatic natural killer cells (NKs)^29^. Upon type III IFN binding to the receptor, a signal transduction cascade ensues involving activation of JAK1, JAK2 and Tyk2, followed by STATs activation and ISG expression, almost identical to that induced by type I IFN receptor^27,30^.

Whilst anti-viral TLRs and RLRs are well recognised for their role in inducing type I and type III IFNs, more recently RNA polymerase III (RNA pol III), an enzyme involved in the transcription of non-coding RNA, was reported to act as an anti-viral PRR by regulating type I IFN induction through generation of a RIG-I ligand^31,32^. RNA pol III is able to transcribe AT-rich dsDNA into the 5′ppp-dsRNA format required for recognition by RIG-I and subsequent IFN induction^32^. Interestingly, an autoantigen associated with systemic autoimmune disease, La/SSB (La), binds to RNA pol III transcripts and stabilises newly-synthesised RNAs^33–38^. In addition to its interaction with a large variety of newly-formed RNAs, La binds a number of virus-encoded RNAs, such as adenovirus VA RNA I and VA RNA II, EBV EBER 1 & 2 RNA, and leader RNA of negative strand RNA viruses^39–42^. Because La can interact with viral RNA, studies have sought to clarify its role in anti-viral immunity. Some studies proposed that La is manipulated by viruses in an attempt to block the anti-viral response, which it reportedly achieves by binding and sequestering the dsRNA ligand for RIG-I, thus preventing activation of the pathway^43–45^. On the other hand, La was also shown to promote an anti-viral response to *flock house* virus (FHV), although the mechanism involved was unclear^46^. Thus the role of La in regulating anti-viral immune responses is not well understood.

Our work described herein demonstrates a novel positive role for La in regulating type I and type III IFN responses downstream of Sendai virus infection. Our results show that knockdown of La severely impairs the ability of cells to mount an anti-viral response to Sendai virus infection, resulting in enhanced infectivity, as a result of reduced type I and III IFN production. We observed that La bound RIG-I in a ligand-inducible manner and that the CARD domains of RIG-I and RNA-binding domain of La are required for this interaction. The association between La and RIG-I promotes the interaction of RIG-I with dsRNA, thereby enhancing RIG-I-driven type I and type III IFN induction. Thus, La is required for an optimum IFN response to Sendai virus infection by binding to the anti-viral RIG-I receptor and promoting its interaction with its cognate ligand.

## Results

### La depletion results in enhanced Sendai virus infection efficiency and decreased Type I and Type III Interferon responses

Sendai virus (SeV) strains are enveloped paramyxoviruses with single-stranded, non-segmented, negative sense RNA genomes. SeV strains may vary significantly in their degrees of virulence. A number of virulence factors have been mapped to either structural proteins associated with differential virus attachment and entry into host cells, or non-structural proteins implicated in immune modulation that includes antagonism of interferon signalling^47–50^. This study utilized a recombinant SeV expressing eGFP (rSeV/eGFP) and SeV *Cantell* strain, both capable of inducing type III IFN responses, while only the *Cantell* strain induces an additional robust type I IFN response^51,52^. Evidence suggests that this differential induction of type I IFN is due to the presence of defective-interfering (DI) particles in the *Cantell* strain^53,54^.

**Figure 1:**
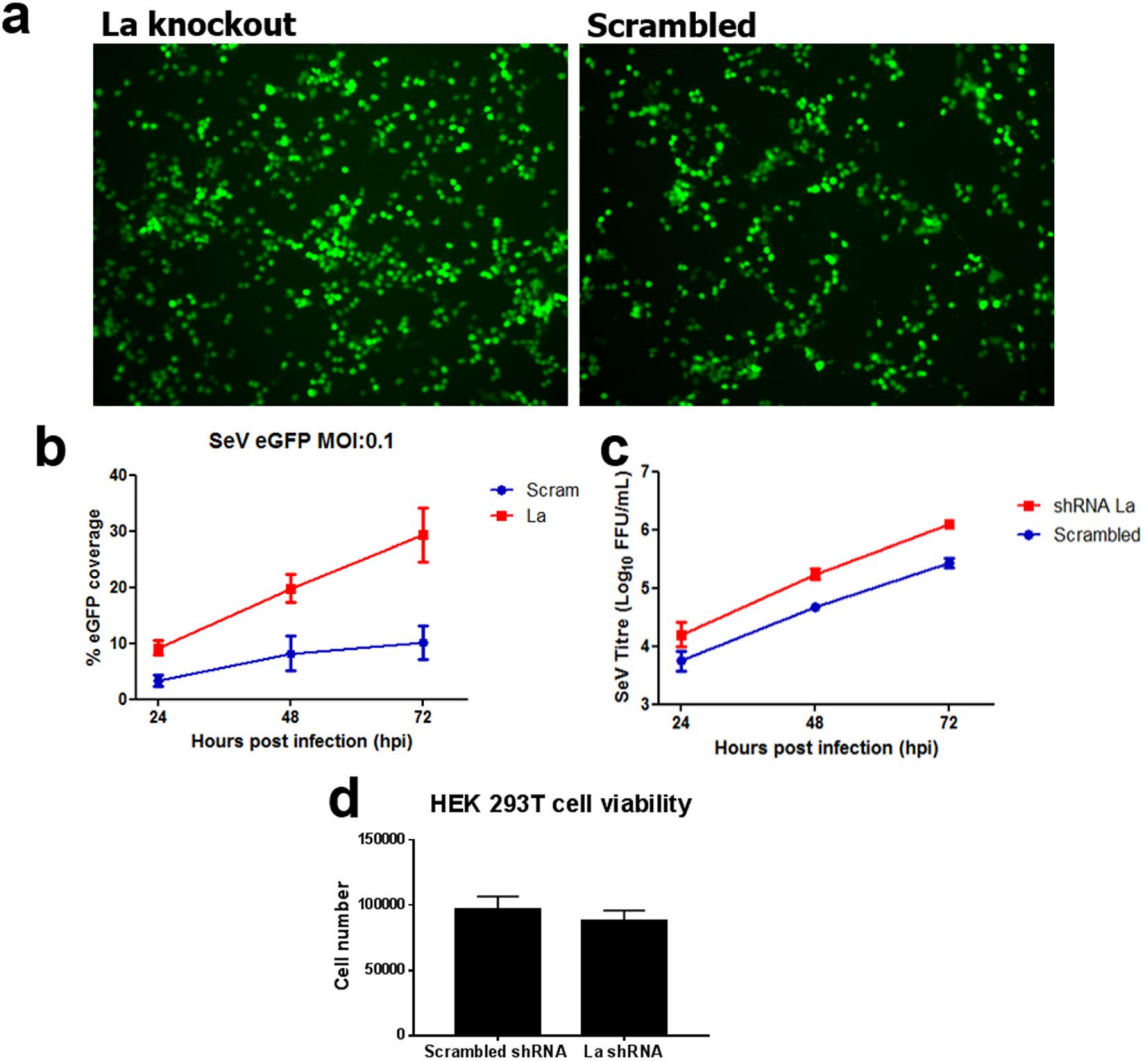
Depletion of La in HEK 293 T results in an increase in Sendai viral infection efficiency. HEK 293 T cells were transfected with 500 ng of La-specific or scrambled Mission® shRNA (Sigma) for 48 h. Cells were then infected with rSeV/eGFP at an MOI of 0.1. (**a**) GFP positivity was visualised using a Nikon Eclipse TE2000-U and a Hamamatsu ORCA ER camera. (**b**) % monolayer GFP-positive analysis was carried out using Image J software. (**c**) rSeV/eGFP titrations (FFU/mL) were carried out on LLC-MK2 cells. Areas under the curves were calculated and compared. Results are from two independent experiments carried out in duplicate. (**d**) 48 h post transfection, a well from each condition (Scrambled or La) was trypsinised, following which a trypan blue cell count was performed to determine the number of viable cells prior to infection. Data shown is combined average cell counts from two independent experiments.

To investigate the effect of La knockdown on Sendai infectivity, HEK 293 T cells infected with rSeV/eGFP and transfected with either a La-specific or scrambled shRNA were evaluated by UV microscopy, using areas of comparable monolayer confluency for image analyses (Fig. 1). Supplemental Fig. 1 demonstrates successful La depletion in HEK 293 T cells, both at gene (Supplemental Fig. 1a and b) and protein (Supplemental Fig. 1c and d) levels, validating the La shRNA construct used throughout this work. Analysis of fluorescence (using Image J software) demonstrated higher eGFP coverage following rSeV/eGFP infection in cells depleted of La compared with those transfected with the scrambled control, suggesting that knockdown of La enhanced viral infectivity (Fig. 1a and b). Importantly, the increased eGFP coverage seen in La-depleted monolayers was reflected in an increase in viral titres released from these cells, compared with control cells (Fig. 1c). As La has been published to be involved in a number of cellular processes, including RNAi processing, the viability of cells transfected with scrambled or La shRNA was compared in order to ensure that La knockdown did not affect cell viability. As shown in Fig. 1d, cell viability was equivalent across the 2 different experimental conditions.

**Figure 2:**
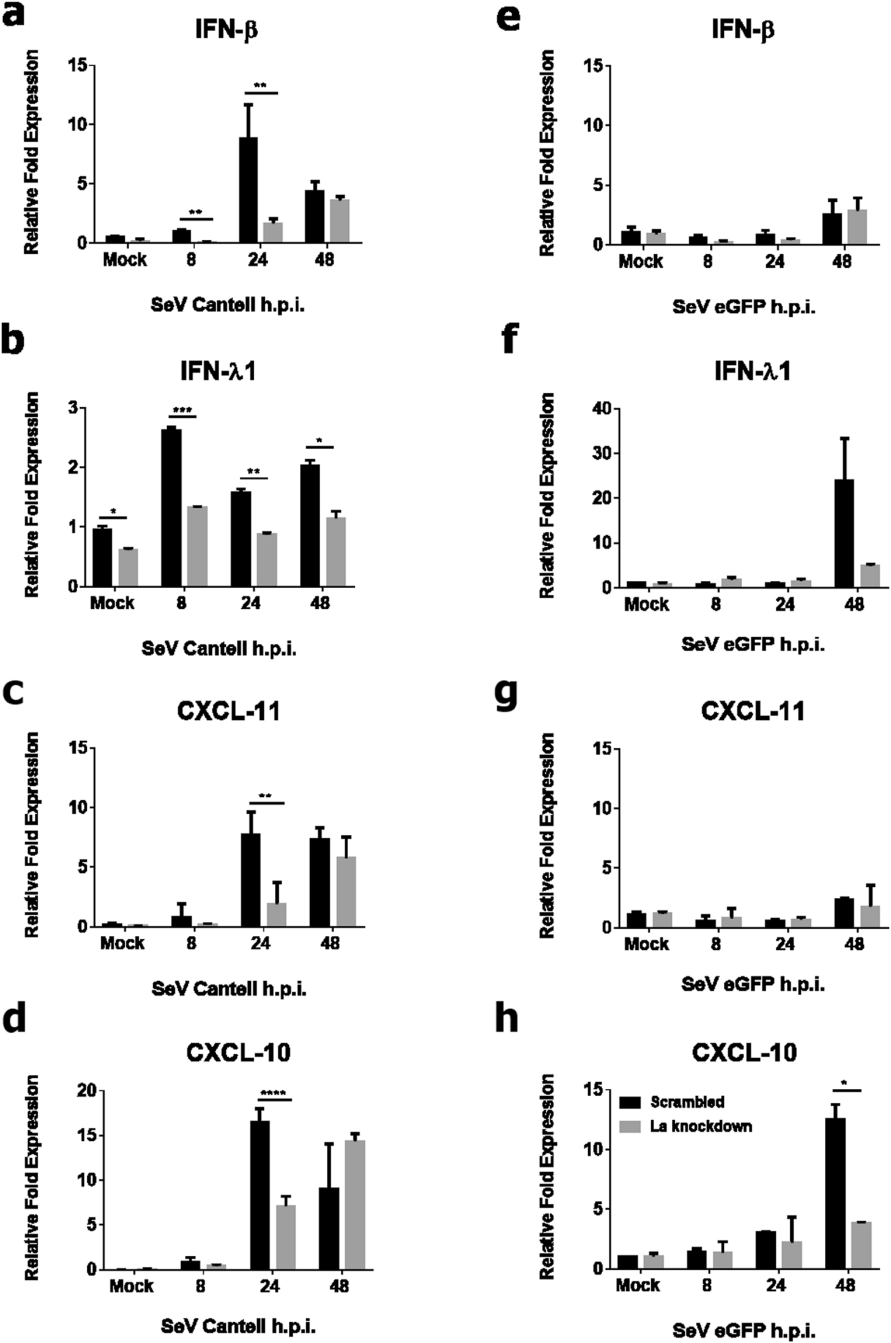
IFN-β, IFN-λ and ISG mRNA expression is attenuated in La-depleted cells following SeV infection. HEK 293 T cells were transfected with 500 ng of either La-specific or scrambled Mission® shRNA (Sigma) for 48 h after which they were infected with SeV *Cantell* (**a**–**d**) or rSeV/eGFP (**e**–**h**) at an MOI of 10 and incubated for the indicated time points. IFN-β (**a,e**), IFN-λ1 (**b,f**), CXCL-11 (**c,g**), CXCL-10 (**d,h**) expression was determined by RT-qPCR. Data shown are a representative of three independent experiments in each case. *p < 0.05, **p < 0.01, ***p < 0.001 and ***p < 0.0001, as determined by unpaired *t*-test, comparing scrambled to La shRNA at each time point.

We next investigated the effect of La knockdown in HEK 293 T cells on type I and type III IFN induction by quantitative PCR (qPCR) following rSeV/eGFP or SeV *Cantell* infection. As stated above, rSeV/eGFP induced a strong type III IFN response but no type I IFN, whereas the SeV *Cantell* induced a robust type I IFN response in addition to type III IFNs^51,52^. La knockdown resulted in significant reduction of both IFN-β and IFN-λ1 mRNA levels following SeV *Cantell* infection (Fig. 2a and b), whereas a reduction in only IFN-λ1 expression was observed in La-depleted cells following infection with rSeV/eGFP, albeit at the later time point of 48 hours post-infection (hpi) (Fig. 2e and f). Importantly, La knockdown had no effect on housekeeping gene expression as shown in Supplemental Fig. 1e. Interestingly, expression of CXCL-10 (IP-10) was significantly attenuated by La depletion following infection with both SeV strains, compared with scrambled controls (Fig. 2d and h). CXCL-11 was only impaired in La-depleted cells following *Cantell* infection (Fig. 2c and g), suggesting that CXCL-11 induction is specifically regulated by La in the context of a type I IFN response. In contrast, the induction of CXCL-11 in response to rSeV/eGFP was poor, with La depletion having no effect on the cells’ ability to mount a response.

**Figure 3:**
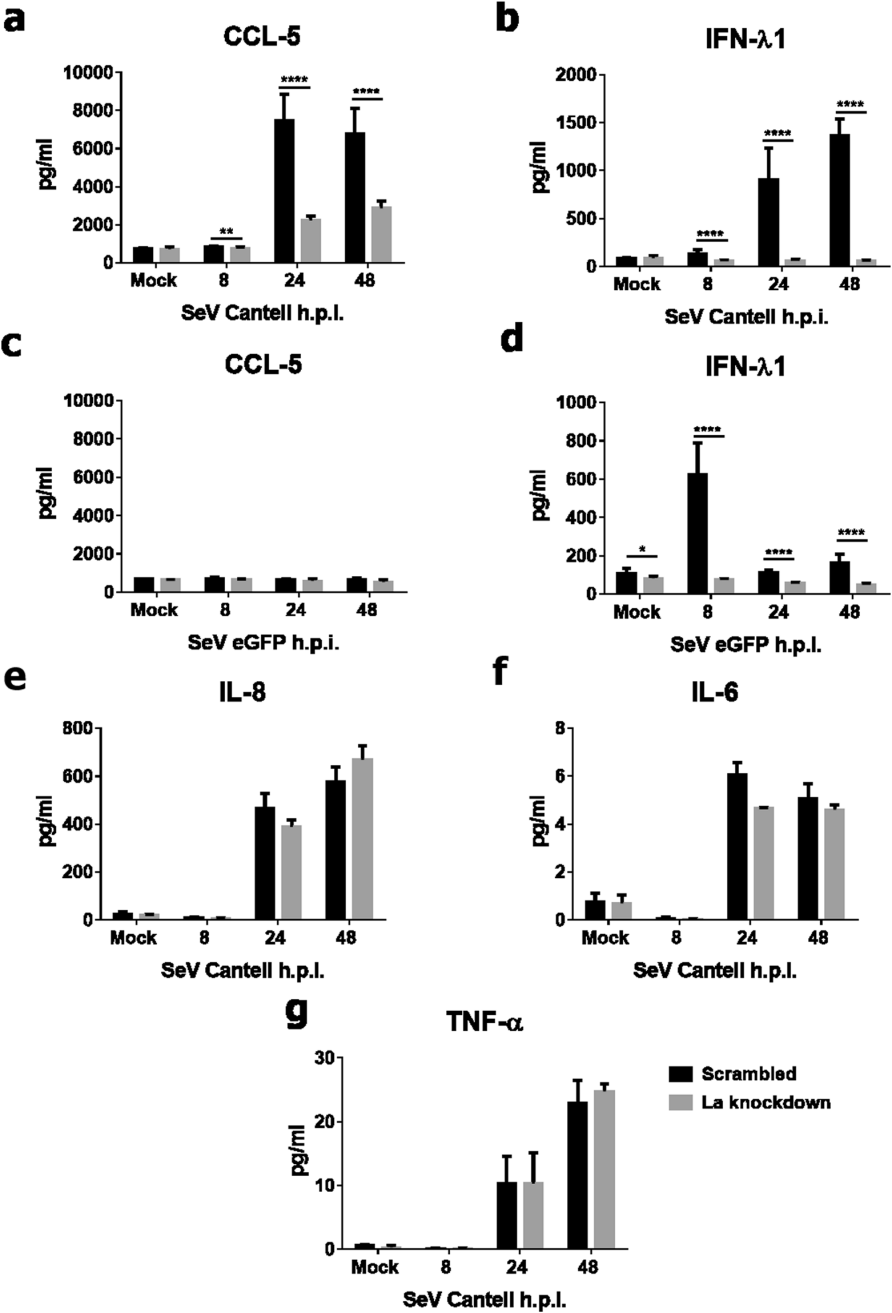
CCL-5 & IFN-λ1 release is decreased in La-depleted cells following SeV infection. HEK 293 T cells were transfected with 500 ng of either La-specific or scrambled Mission® shRNA (Sigma) for 48 h after which they were infected with SeV *Cantell* (**a,b**) or rSeV/eGFP (**c,d**) at an MOI of 10. CCL-5 (**a,c**) and IFN-λ1 (**b,d**) cytokine release was determined by ELISA. IL-8 (**e**), IL-6 (**f**) and TNF-α (**g**) was measured from cells supernatants using a multi-plex human pro-inflammatory 7-spot assay (MSD). Data shown is the combined average of three independent experiments. *p < 0.05, **p < 0.01 and ****p < 0.0001, as determined by unpaired *t*-tests, comparing scrambled to La shRNA at each time point.

Analysis of cytokine release following SeV infection demonstrated that CCL-5 (RANTES, a type I and III-regulated chemokine) production was impaired following SeV *Cantell* infection in La-depleted cells (Fig. 3a). Unsurprisingly, given that ISG expression was only induced at mRNA level following 48 hpi, no CCL-5 release was detected following infection with rSeV/eGFP strain across the 48 hour time course of infection, nor was any significant difference observed in La-depleted cells, compared with scrambled controls (Fig. 3c). IFN-λ1 release was completely abrogated in La-depleted cells, compared with controls, following infection with either SeV strain, further supporting our findings suggesting that La is crucial for the IFN response downstream of viral infection (Fig. 3b and d). Assessing the effect of La knockdown on proinflammatory cytokine production in response to SeV *Cantell* infection demonstrated that La knockdown had little or no effect on the ability of the *Cantell* strain to induce IL-8, IL-6 or TNF-α (Fig. 3e–g). Importantly, our results demonstrate not only that La positively regulates type I IFN responses downstream of SeV infection, but that it also has a novel role in promoting type III I IFN induction downstream of SeV infection.

### La enhances RIG-I binding to RNA ligand via direct interaction with the CARD-domain of RIG-I

**Figure 4:**
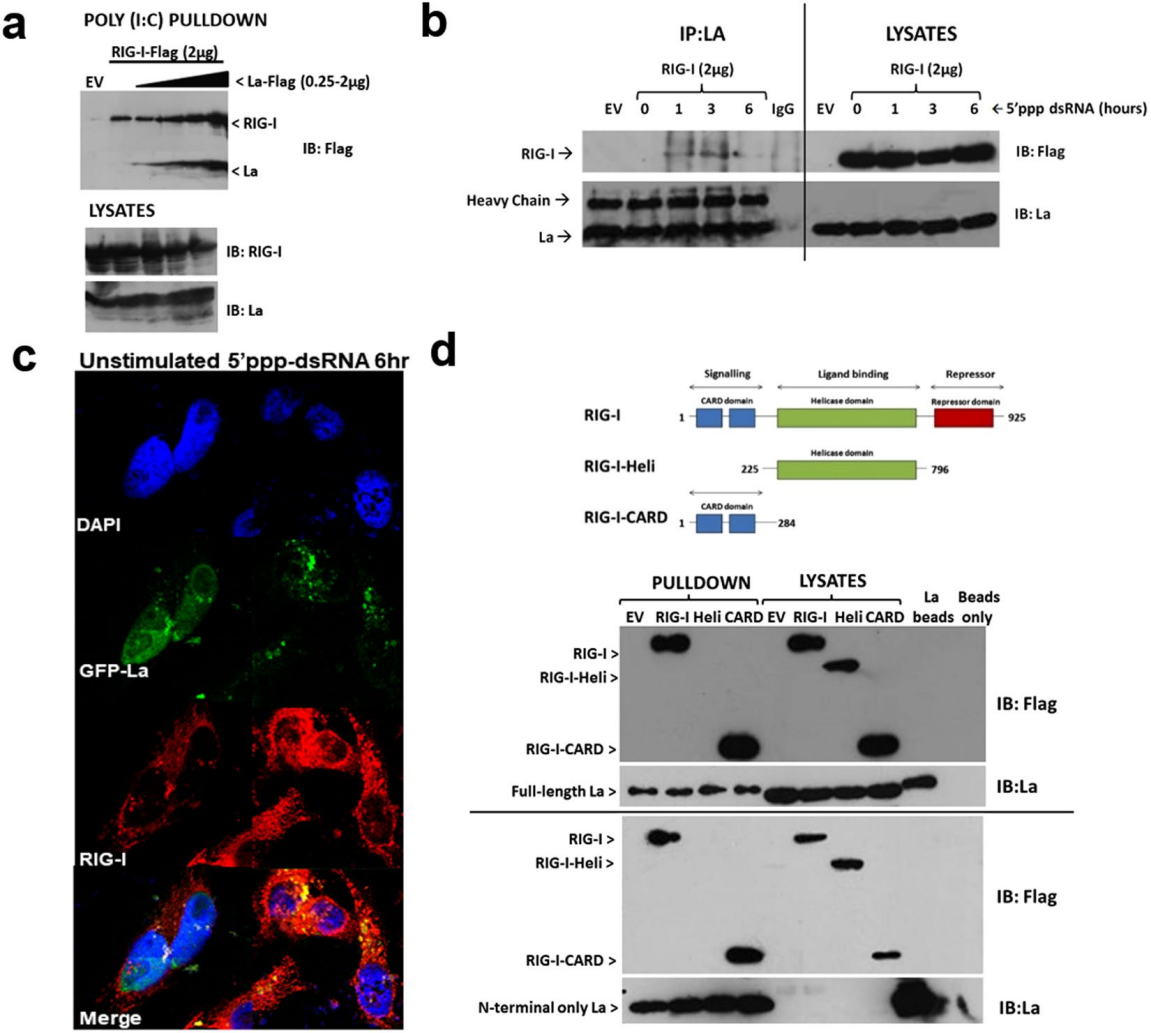
La enhances RIG-I binding to RNA ligand and interacts with RIG-I following 5′ppp-dsRNA stimulation. (**a**) HEK 293 T cells were transfected with 4 μg EV or 2 μg FLAG-tagged RIG-I with increasing FLAG-tagged La, as indicated. Analysis of the ability of FLAG-tagged La or RIG-I to bind biotinylated poly(I:C) was assessed by western blotting with anti-FLAG antibody. Expression of FLAG-tagged or RIG-I constructs in whole cell lysates was determined by western blotting with either anti-La or anti-RIG-I antibodies, as appropriate. (**b**) HEK 293 T cells were transfected as indicated and stimulated for 1, 3, or 6 h with 1 μg 5′ppp-dsRNA (Invivogen). Following immunoprecipitation of La-containing complexes with a La-specific antibody, the ability of over-expressed RIG-I to interact with endogenous La was determined by western blotting using anti-FLAG antibody. (**c**) HeLa cells were seeded on UV-irradiated coverslips, transfected with 2 μg of GFP-tagged La and 2 μg FLAG-tagged RIG-I, following which they were stimulated with 1 μg 5′ppp-dsRNA for 6 h. Immunostaining with anti-RIG-I antibody indicates that La and RIG-I co-localise following stimulation with 5′ppp-dsRNA. Both images are at 63X magnification. (**d**) Recombinant La was incubated with lysates prepared from HEK 293 T cells overexpressing FLAG-tagged RIG-I, FLAG-tagged RIG-I-CARD, or FLAG-tagged RIG-I-Helicase (Heli), as indicated. The ability of RIG-I and La to interact was analysed by western blotting. In all cases images are representative of three independent experiments.

SeV *Cantell* has been reported to rely entirely on RIG-I to elicit an anti-viral immune response^55^. Having demonstrated that La is required for type I and III IFN production in response to SeV challenge, and given its ability to bind RNA, we hypothesised that La may directly regulate RIG-I activation through regulation of RNA binding. To test this hypothesis, HEK 293 T cells were transfected with FLAG-tagged RIG-I and increasing concentrations of La from 0–2 μg. Cell lysates were incubated with 1 μg biotin-labelled poly(I:C), and poly(I:C)-binding proteins were subsequently isolated. The ability of RIG-I to bind poly(I:C) was determined by western blotting using anti-FLAG antibody. As Fig. 4a shows, increasing concentrations of La enhanced the ability of RIG-I to bind poly(I:C). A faint lower band on the gel (corresponding to La) indicates, as would be expected from previous reports, that La was also capable of direct interaction with the RNA ligand. The lower panel of Fig. 4a demonstrates total RIG-I and La expression in the lysates; endogenous La can be observed strongly in the lane without FLAG-tagged La overexpressed, due to blotting with anti-La antibody. However, transfection with increasing concentrations of La (lanes 2–5) dose-dependently increases expression, as expected. Full blots as well as statistical analysis of corresponding optical densitometry across three individual experiments are shown in Supplemental Fig. 2.

Having observed enhanced binding between RIG-I and its RNA ligand in the presence of over-expressed La, we hypothesised that La may achieve this through direct binding to RIG-I. Co-immunoprecipitation studies demonstrated an inducible interaction between La and RIG-I following stimulation of RIG-I overexpressing HEK 293 T cells with the RIG-I agonist, 5′ppp-dsRNA (Fig. 4b, Supplemental Fig. 3). This inducible interaction was statistically significant across three independent experiments (Supplemental Fig. 3d). In addition, HeLa cells over-expressing GFP-La and flag-tagged RIG-I were stimulated with 5′ppp-dsRNA, which induces La translocation from the nucleus to the cytoplasm where it can co-localise and interact with RIG-I (Fig. 4c). Additional confocal images demonstrating this pattern are shown in Supplemental Fig. 4 and overlap coefficients for each image are given in Supplemental Table S4.1. The translocation of La from the nucleus to the cytoplasm is consistent with data from previous studies that demonstrate similar translocation of La following viral infection^44,45,56^. In addition, cells depleted of La had a reduced response to 5′ppp-dsRNA in their ability to induce IFN-β, confirming the ability of La to directly promote RIG-I induced IFN-β expression (Supplemental Fig. 3c).

In order to determine the domains responsible for this interaction, either full-length or N-terminal-only (aa 1–204) His-tagged recombinant La was incubated with lysates from HEK 293 T cells over-expressing full-length RIG-I, the CARD domain-only of RIG-I, or the helicase domain-only of RIG-I. Potential interactions were analysed by western blotting. As Fig. 4d (upper panel) demonstrates, an interaction was observed between full-length La and full-length RIG-I, as expected, but also with the RIG-I deletion mutant expressing only the CARDs. No interaction was observed with helicase-only mutant of RIG-I. This indicates that the activatory CARD domains (spanning amino acids 1–284) of RIG-I are necessary and sufficient for the interaction between La and RIG-I to occur. Further analysis demonstrated that the N-terminal but not the C-terminal domain of La is required for interaction with both full length and CARD domains of RIG-I (Fig. 4d, lower panel).

Collectively, our results indicate a novel role for La as a positive regulator of type I and type III IFN production in response to SeV infection. We demonstrate that the mechanism of this regulation occurs through a direct interaction between La and RIG-I, which promotes RIG-I binding to its cognate ligand. Our findings not only contribute to the understanding of molecular mechanisms behind RIG-I-mediated regulation of IFN induction, but also provide valuable insight into the potential that dysregulation of La activity may contribute to over-activation of RIG-I and hence dysregulated IFN production, as observed in autoimmune diseases such as SLE.

## Discussion

The induction of IFN expression is a crucially important part of the innate anti-viral immune response, not only for destruction of viral RNA and limitation of viral spread, but also for activation of adaptive immunity and selective killing of virally-infected host cells. With this work we have demonstrated a novel interaction between La and RIG-I, which results in enhanced RIG-I-RNA association. Knockdown of La resulted in increased Sendai viral infection efficiency, decreased IFN-β, IFN-λ1 and ISG mRNA expression and attenuated CCL-5 and IFN-λ1 release, compared with control cells. Overall, these findings highlight an essential and novel role for La in mediating optimal type I and type III IFN responses following viral challenge in order to protect the host by both limiting viral replication and promoting the clearance of the pathogen.

Type I IFNs are the first line of defence against most types of viral infection, including the murine pathogen, SeV (DI^+^). They induce an anti-viral state in host cells. This is achieved by JAK/STAT pathway-mediated activation of interferon-stimulated genes (ISGs), such as RIG-I, CXCL-10, CXCL-11, OAS1 and OAS2^57^. Our findings demonstrated a significant decrease in the induction of IFN-β, CXCL-10, and CXCL-11 following SeV infection upon depletion of La. While type III IFNs are structurally and genetically distinctive from type I IFNs and act through a separate receptor system, they have similar mechanisms of induction, signal transduction and biological function^26,58^. SeV is a potent inducer of type III IFN responses^27,59^. Our study identifies La as a novel positive regulator of IFN-λ1 induction downstream of SeV infection. Collectively our results indicate an important role for La in inhibiting SeV replication by promoting both type I (as seen following *Cantell* infection) and type III (as seen following *Cantell* and rSeV/eGFP) IFN responses.

RIG-I is central to the regulation of both type I and type III IFN production as it is responsible for detection of SeV infection within cells. Regarding regulation of RIG-I activity, a number of proteins have been identified to play a role through post-translational modifications. For example, TRIM25 and Riplet/RNF135/REUL induce K63-linked ubiquitination within the CARD domains of RIG-I following viral infection, a modification which is necessary for interaction with IPS-1^60–62^. In addition, CK2-mediated phosphorylation of RIG-I at Thr 770 and Ser 854 inhibits the anti-viral response to both hepatitis C virus and SeV and renders RIG-I inactive^63^. This prevents TRIM25-mediated ubiquitination of RIG-I, thereby negatively regulating the IFN response^60^. With this work, we have identified a novel function for the autoantigen La in enhancement of anti-viral responses. Specifically, it binds directly to the RIG-I receptor in an inducible manner and strengthens RIG-I binding to its RNA ligand, making it unique in its mechanism of action from other known RIG-I regulators, such as TRIM25 and CK2. Thus La positively regulates type I and type III IFN responses by augmenting stable RNA-RIG-I complex formation, which results in robust pathway activation. As RIG-I can also drive inflammatory gene expression through interaction with a IPS-1-CARD9-Bcl-10 complex and activation of NFκB^64^, it would appear that the interaction between La and RIG-I is able to enhance the IFN-β response (presumably via enhancing interaction of IPS-1 with TBK-1) possibly independent of the ability of RIG-I to drive NFκB activation. This is supported by the fact that La knockdown has no effect on inflammatory gene expression downstream of RIG-I interaction, whereas IFN-β expression is severely reduced.

Regarding a potential role for La in regulating assembly of RNA-binding complexes, Liu and colleagues^46^ demonstrated a role for La in RNAi processing. They reported that La associated with Ago2 of the RISC complex in an RNA-dependent manner, thereby promoting RISC complex catalysis and RNAi processing. This finding is consistent with our data which show that La augments RIG-I binding to poly (I.:C). Importantly, deletion of the RNA-binding domain of La blocks interaction with RIG-I, underlining the RNA-binding role of La in driving RIG-I activity. In keeping with our findings that La is a positive regulator of viral-induced type I IFN, Liu *et al*. showed that La could promote the anti-viral response to *flock house* virus (FHV) in Drosophilia S2 cells. Indeed, La depletion resulted in increased FHV infectivity, which supports our findings that La promotes anti-viral responses to Sendai virus in HEK 293 T cells. In contrast, Bitko and colleagues demonstrated enhanced IFN-β mRNA levels and decreased viral titres upon siRNA depletion of La^43^. In addition, Domitrovich *et al*. argued a role for La as a negative regulator of IFN production in the context of HCV replication, based on a 63–67% reduction in RNA replication in the absence of La in Huh7 cells and increased IFN-β mRNA 10 hours post-RSV infection in the absence of La^44^. However, similar to our findings, both of these studies also observed an overall decrease in IFN-β production 24 hours post-infection in La-depleted cells, suggesting that La may play a dual role in regulating anti-viral responses. Indeed, La may play a role in maintaining homeostasis in cells with respect to IFN-β production, as evidenced by the enhanced IFN-β expression in unstimulated cells depleted of La (Supplementary Figure 3c), whereas when RIG-I is activated, depletion of La results in substantial reduction in IFN-β production. This indicates that the loss of La may somehow disrupt homeostatic mechanisms to maintain appropriate IFN-β levels or indeed may inhibit viral-specific evasion mechanisms. For example, the RSV-derived NS2 protein binds to RIG-I and blocks its interaction with IPS-1, thereby preventing IRF-3 activation^65,66^. Thus the loss of La may disrupt the negative function of NS2 on this system, thereby contributing to the enhanced IFN-β observed in similar studies. Extensive studies would be required to address these questions, which are outside the scope of this manuscript.

With this work, we identify a role for La in regulating IFN responses by promoting the RIG-I-mediated anti-viral response through direct association with RIG-I and enhancing RIG-I binding to its viral agonist. Importantly, our study is the first to assess the role for La in regulating type III IFN responses, with all previous studies focusing on type I IFN only. SeV infection experiments support these findings, with depletion of La resulting in increased viral infectivity and decreased type I and type III IFN responses, compared with controls. These findings highlight an important and novel role for La in the promotion of optimal type I and type III IFN responses following SeV challenge, serving to protect the host through limiting viral replication.

## Methods

### Materials

The SW5 monoclonal La antibody was generated by Professor Michael Bachmann at the Technical University of Dresden and was a kind gift from Dr. JS Maier-Moore^67^. All flag-tagged RIG-I plasmid constructs were a kind gift from Dr. Kate Fitzgerald (UMASS Med School, Worcester, MA). The GFP-tagged La construct was a gift from Dr. Karl Albert Brokstad (University of Bergen, Germany). Monoclonal M2 Flag antibody was purchased from Santa Cruz, pcDNA3.1 empty vector control from Invitrogen and biotin-labelled poly(I:C) from Cayla-Invivogen. A Mission® shRNA construct specific to human La, as well as a scrambled control, were purchased from Sigma.

### Cell Culture

HEK 293 T and HeLa cell lines were cultured in Dulbecco’s Modified Essential Medium (DMEM) containing stable 2 mM L-glutamine, 10% (v/v) foetal calf serum (FCS), 100 units/ml Penicillin, 100 μg/ml Streptomycin and 100 μg/ml gentamicin. LLC-MK2 cells (ECACC 85062804) were grown in minimum Eagle’s medium (MEM) containing 40 g/ml non-essential amino acids and supplemented with 10% heat inactivated FCS, 2 mM L-glutamine, 2 mg/ml sodium carbonate, 100 g/ml gentamicin and 1.25 mg/ml Fungizone. Cells were maintained at 37 °C in a humidified atmosphere of 5% CO_2_.

### La Knockdown

HEK 293 T cells were seeded at 5 × 10^4^ cells per ml and transfected the following day with 500 ng of scrambled or La-specific shRNA (Sigma). Following 48 hr, cells were washed with PBS prior to viral infection as detailed below.

### Viral Infection

The rescue and characterisation of recombinant Sendai virus expressing eGFP (rSeV/eGFP) was previously described^68^. The SeV Cantell strain, a wild type strain containing defective interfering particles, comes originally from Charles River Laboratories. Media was discarded and replaced with fresh pre-warmed DMEM supplemented with antibiotic only (no FCS), in order to limit cell growth. Cells were then infected with either rSeV/eGFP or SeV *Cantell* at a multiplicity of infection (MOI) of 0.1 or 10, as indicated in figure legends. One hour post-infection, inocula were removed by discarding media and replaced with DMEM supplemented with antibiotic and 1% FCS to ensure cell survival while maintaining limited growth. At indicated time points, media was carefully removed and retained for subsequent cytokine analysis, cells were gently re-suspended in ice-cold PBS and centrifugation was carried out at 400 × *g* for 5 min to pellet cells for subsequent analysis. Cells were then re-suspended in Trizol reagent for gene expression analysis or SDS sample buffer supplemented for protein expression analysis.

### SeV titrations

For SeV/eGFP, a 1:10 dilution series of the sample was added to LLC-MK2 cells in MEM 1% FBS. At 24 hpi fluorescent foci were counted. The titer is calculated as fluorescent forming units (FFU) by multiplying the average number of foci by the dilution factor at a given dilution. The dilution at which the foci were counted is equal to the inverse of the exponent of the final FFU. The titer of SeV Cantell stock was determined by plaque assay as previously described^69^.

### Real-time polymerase chain reaction (qPCR)

RNA was extracted from cell cultures using Trizol™ (Sigma) and reverse transcribed to complementary DNA using the GoScript Reverse Transcription kit (Promega), as per manufacturer’s instructions. Real-time quantitative PCR investigating gene expression was performed using primers listed in Table 1, with SYBR Green Taq ReadyMix (Sigma) according to manufacturer’s recommendations. Data were analyzed using an ABI Prism 7900 system (Applied Biosystems) and were normalized to 18 s RNA. Real-time PCR data were analyzed using the 2^−ΔΔct^ method^70^.

**Table 1:** Human primers used in this study.

Primer Name	Primer Sequence	Product Size(bp)
La sense	GAAGGAGAGGTGGAAAAAG	372
La anti-sense	AAGCCCCGCAAACAAAAG	
IFN-β sense	CTAGCACTGGCTGGAATGAGA	217
IFN-β anti-sense	CTGACTATGGTCCAGGCACA	
18 S sense	TTGACGGAAGGGCACCACCA	131
18 S anti-sense	GCACCACCACCCACGGAATCG	
IFN-λ1 sense	GGACGCCTTGGAAGAGTCACT	84
IFN-λ1 anti-sense	AGAAGCCTCAGGTCCCAATTC	
CXCL-10 sense	GGAAGCACTGCATCGATTTTG	519
CXCL-10 anti-sense	CAGAATCGAAGGCCATCAAGA	
CXCL-11 sense	GCCTTGGCTGTGATATTGTGTG	686
CXCL-11 anti-sense	CACTTTCACTGCTTTTACCCCAG	

### Western blotting

To prepare whole cell lysates, cells were lysed in SDS buffer (250 mM Tris-HCl, pH 6.8, 10% SDS, 0.5% Bromophenol blue, 50% Glycerol, 50 nM DTT) and boiled at 95 °C for 10 min. Equal quantities of whole cell lysates were resolved by electrophoresis on a denaturing SDS–polyacrylamide gel according to the method of Laemmli^71^ and transferred to a nitrocellulose membrane. Following immunoblotting, the membrane was developed using enhanced chemiluminescent horse radish peroxidase (HRP) substrate (Millipore).

### Co-immunoprecipitation

Cells were treated as described in the figure legends, lysed in EBC lysis buffer (Deionised water containing 50 mM Tris (pH 8.0), 150 mM NaCl, 1% Nonidet P40, 0.5% (w/v) sodium deoxycholate and 0.1% SDS containing 1 mM Sodium orthovanadate (Na_3_VO_4_), 1 mM Phenylmethylsulfonylfluoride (PMSF), 1 mM Potassium fluoride (KF),) and incubated with SW5 anti-La antibody coupled to protein A sepharose beads. Thereafter, immune complexes were washed and re-suspended in SDS sample buffer for western blot analysis.

### Recombinant protein pull-downs

Following a gentle wash with ice-cold PBS, lysates were prepared by addition of EBC buffer. After sonication and centrifugation, the supernatant was incubated with 50 μl Nickel agarose beads coupled to approximately 1 μg either full length (8 A) or N-terminal truncated (7 A) recombinant La (Dr. J. Maier-Moore), for 2 h on rotation at 4 °C. After incubation, nickel agarose was washed three times with EBC buffer by gentle inversion and centrifugation at 5,000 × *g*. Beads were then re-suspended in SDS sample buffer for western blot analysis.

### Enzyme-linked Immunosorbance Assay (ELISA)

ELISAs were carried out using DuoSet® ELISA Development Kit for human CCL-5 (Rantes) or human IFN-λ1 (IL-29) (eBioscience) as per the manufacturer’s instructions.

### RNA Immunoprecipitation

Cells were seeded and transfected as indicated in figure legends. Cells were lysed for 20 min at 4 °C on rotation in freshly prepared sterile RNA Immunoprecipitation (RIP) buffer (150 mM KCl, 25 mM Tris pH 7.4, 5 mM EDTA, 0.5 mM DTT, 0.5% NP-40) supplemented with protease inhibitors and SUPERase RNAse inhibitor (Sigma). Samples were sonicated for 15 sec, cell debris was pelleted by centrifugation and cell supernatants were transferred to fresh tubes. One µg of biotin-labelled poly(I:C) (Invivogen) was added, followed by incubation at 4 °C for 1–2 h. The samples were then added to 50 µl of pre-washed UltraLink NeutrAvidin beads (Pierce) and incubated for 1 h at 4 °C. The resulting immune complexes were then washed with RIPA buffer and re-suspended in 30 µl SDS sample buffer, prior to western blot analysis.

### Immunofluorescence

HeLa cells were seeded at 1 × 10^5^/well on coverslips, transfected and stimulated as indicated in figure legends. Cells were fixed and permeabilised with 4% paraformaldehyde and 0.2% (v/v) Triton X-100 in PBS. After washing, cells were blocked in PBS with 1.2% (w/v) Fish Gelatin and 100 mM glycine and then incubated at 37 °C for 1 h with the primary antibody of interest at 1:100 dilution in blocking buffer, followed by detection with the appropriate fluorescently labelled secondary antibody at 1:200 dilution. Cells were mounted and nuclei stained using ProLong® Gold anti-fade reagent with DAPI. Cells were imaged using the LSM 710 System (Carl Zeiss) and analysed for co-localisation using Zen 9 software.

### Statistical analysis

All data was analysed using GraphPad Prism (version 7) statistical software package, as specified. Statistical comparison between groups was carried out using tests described in figure legends. Data is graphically represented as mean +/− standard error of the mean (SEM). *P* values less than or equal to 0.05 were considered significant.

### Data Availability

• No datasets were generated or analysed during the current study.

• All data generated or analysed during this study are included in this published article (and its Supplementary files).

## Electronic supplementary material

Supplementary Data
