# Supplementary material for: The RNA binding protein La/SS-B promotes RIG-I-mediated type I and type III IFN responses following Sendai viral infection
Source: Sci Rep. 2017 Nov 6;7:14537. doi: 10.1038/s41598-017-15197-9 (PMC5673980; doi:10.1038/s41598-017-15197-9)
Supplement: Supplementary file 1 — Supplementary Data [file 41598_2017_15197_MOESM1_ESM.pdf]

Supplementary Information

Title

The RNA binding protein La/SSB promotes RIG-I-mediated type I and type III IFN responses following Sendai viral infection

Authors

Rebecca Mahony (1), Lindsay Broadbent (2), Jacen S. Maier-Moore (3), Ultan F. Power (2) and Caroline A. Jefferies (1, 4).

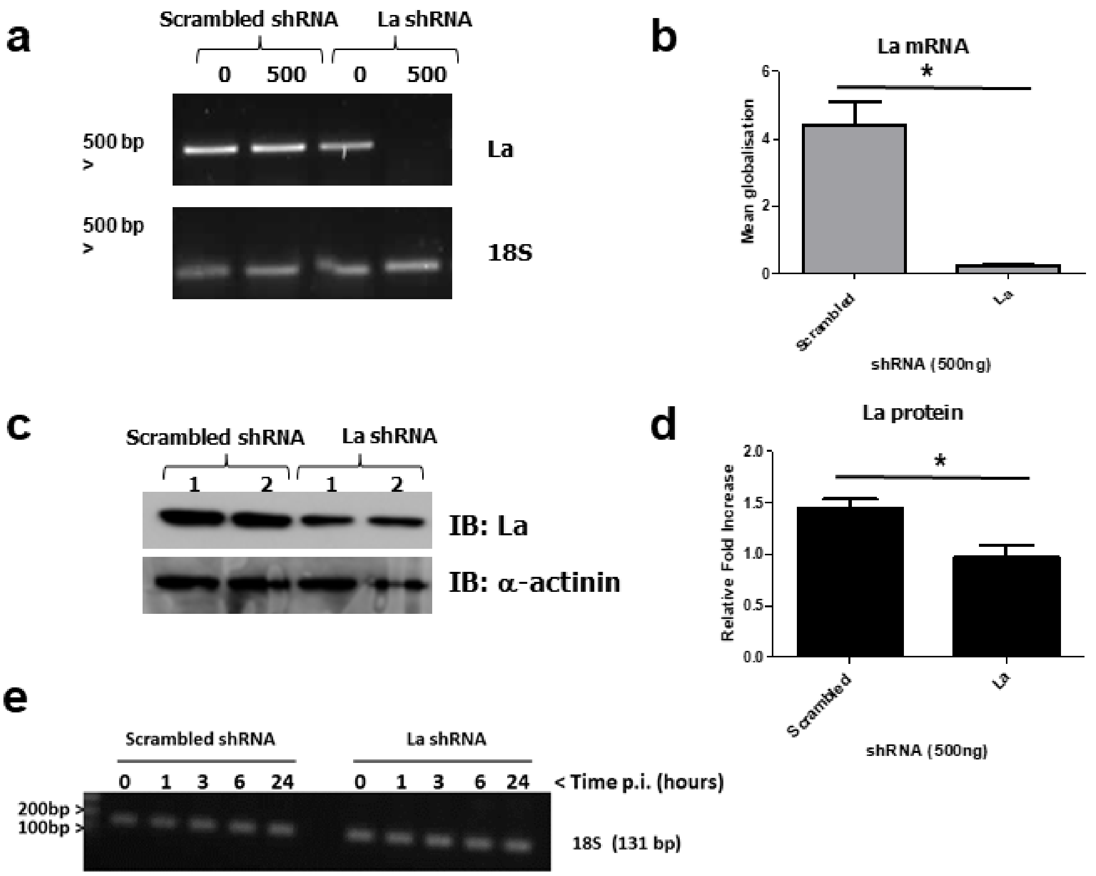

Supplemental Figure 1; Confirmation of La-specific shRNA knockdown and lack of effect of La depletion on house-keeping gene expression: (a) HEK 293T cells were seeded at 5×10^4 cells per ml and transfected with 500 ng of scrambled or La-specific shRNA. Following 48 hr, cells were washed with PBS, RNA was extracted, cDNA was prepared and qualitative PCR and (B) real-time PCR were carried out for La and 18S. (c) HEK 293T cells were seeded at 5×10^4 cells per ml and transfected with 500 ng of scrambled or La-specific shRNA. Following 48 hr, cells were washed, lysed in 5 × sample buffer supplemented with DTT (50 nM) and boiled at 95°C for 5 min. Samples “1” and “2” above represent two independent experiments assessed for indicated proteins on the same western blot. (d) Optical densitometry combined from n=4 of La protein levels normalised to α-actinin protein levels. *$p<0.05$ as determined by unpaired t-test using GraphPad Prism. (e) HEK 293T cells were transfected with 500 ng of either La-specific or scrambled Mission® shRNA (Sigma) for 48 h after which they were infected with SeV *Cantell* (MOI 10) for indicated time course (p.i. – post-infection). Cells were then harvested and 18S gene expression was measured using qualitative PCR and agarose gel electrophoresis.

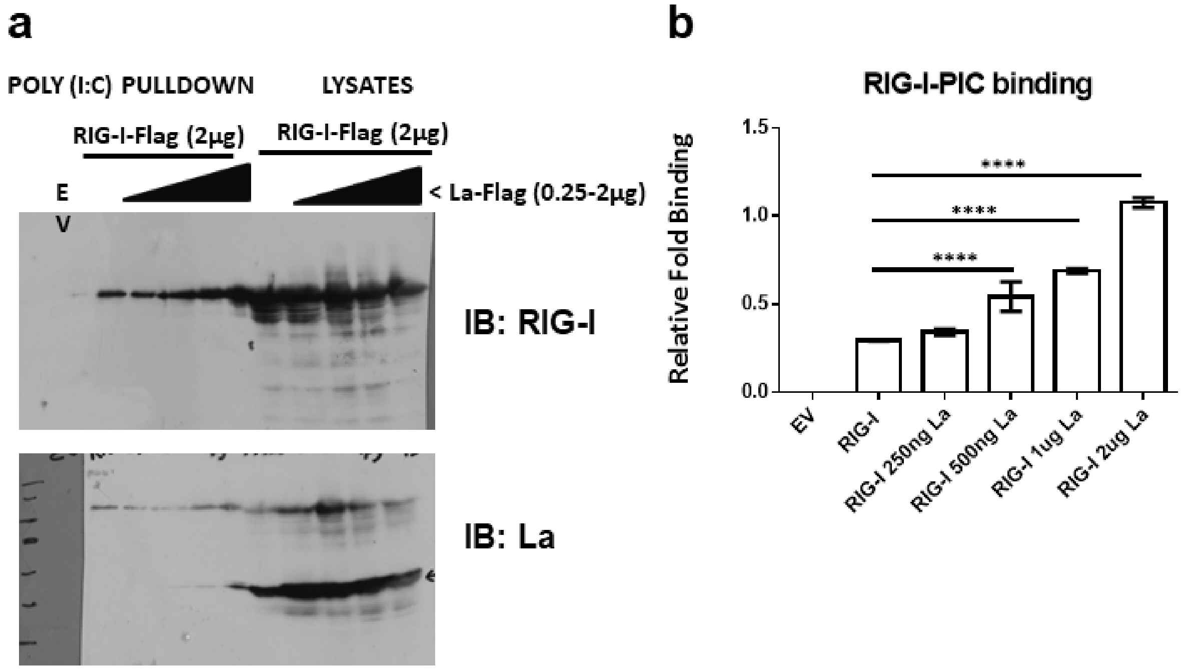

Supplemental Figure 2; RIG-I binding to RNA ligand, Poly(I:C), is enhanced by La overexpression (a) HEK 293T cells were transfected with 4 µg EV or 2 µg FLAG-tagged RIG-I with increasing FLAG-tagged La, as indicated. Analysis of the ability of La (lower panel) or RIG-I (upper panel) to bind biotinylated poly(I:C) was assessed by western blotting. (b) Optical densitometry carried out on RIG-I IP bands, relative to RIG-I bands in the lysates, Dunnett's multiple comparison test was carried out using Graphpad Prism, ****$p<0.0001$

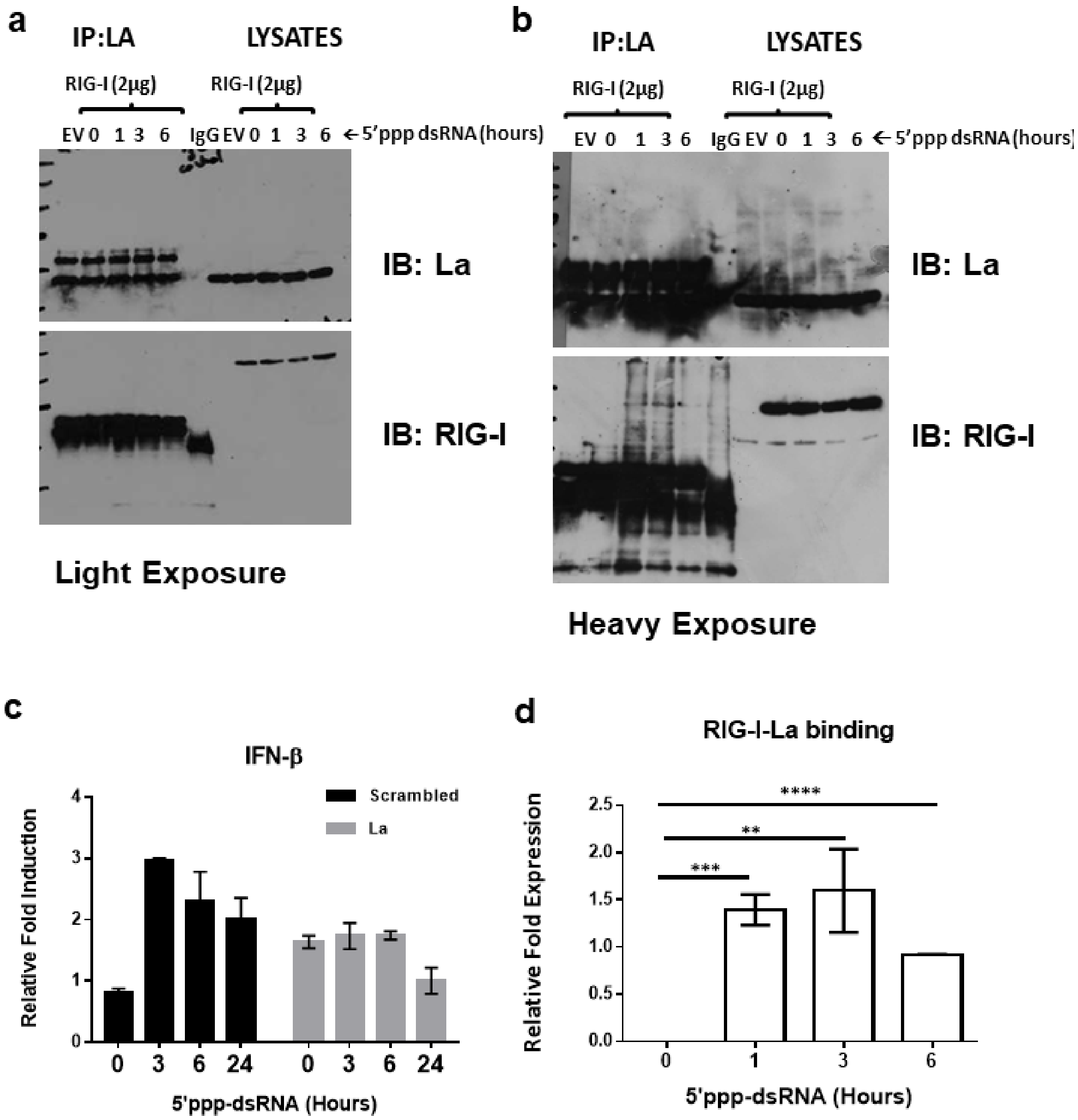

Supplemental Figure 3; 5'ppp-dsRNA stimulation leads to RIG-I-La interaction and IFN-β gene induction: HEK 293T cells were transfected with empty vector (lanes 1, 7) or RIG-I (lanes 2-5, 8-11) as indicated and stimulated for 1h (lanes 3 & 9), 3h (lanes 4 &10), or 6h (lanes 5 &11) with 1 μg 5'ppp-dsRNA (Invivogen). The SW5 anti-La antibody was coupled to agarose beads and used to pull down endogenous La protein in order to immunoprecipitate (IP) any potential interactors in HEK293T lysates. IP samples are in lanes 1-5, IgG control in lane 6 and corresponding lysates in lanes 7-11. Successful pulldown was determined by western blotting for La (upper blots). The ability of over-expressed RIG-I to interact with endogenous La was determined by western blotting for RIG-I-flag (lower blots). Blots shown represent (a) light and (b) heavy exposure of the same membranes. (c) HEK 293T cells were transfected with 500 ng of either La-specific or scrambled Mission® shRNA (Sigma) for 48 h after which they were transfected with 1μg 5'ppp-dsRNA (Invivogen) at indicated time points. IFN-β expression was determined by RT-qPCR. Data shown are triplicate values representative of an experiment that was repeated twice. (d) Optical densitometry carried out on panel (b), $^{**}p<0.01$, $^{***}p<0.001$, $^{****}p<0.0001$, as determined by unpaired *t*-tests.

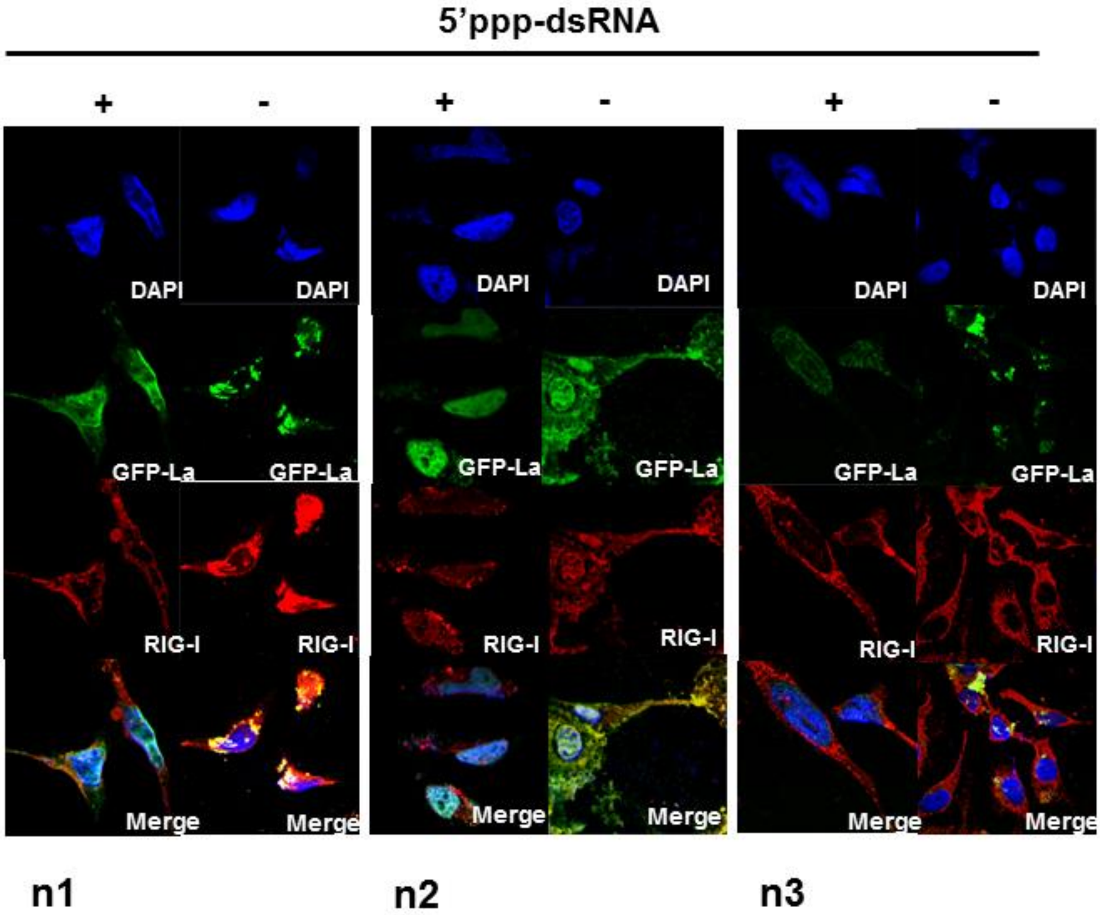

Supplemental Figure 4; 5’ppp-dsRNA stimulation leads to RIG-I-La co-localisation: HeLa cells were seeded on UV-irradiated coverslips, transfected with 2 µg of GFP-tagged La and 2 µg FLAG-tagged RIG-I, following which they were stimulated with 1 µg 5’ppp-dsRNA for 6 h. Immunostaining with anti-RIG-I antibody indicates that La and RIG-I co-localise following stimulation with 5’ppp-dsRNA. All images are representative of multiple images taken from three independent experiments and are at 63X magnification.

Table S4.1: Overlap coefficient analysis for Fig4c & Supplemental Fig4; Co-localisation analysis was performed on the total area of each confocal image presented using Zen 9 software (Carl Zeiss), numbers represent the overlap coefficient. An overlap coefficient of 1 represents perfect colocalisation

	Unstimulated	5’ppp-dsRNA
Fig 4c	0.47	0.76
n1	0.56	0.81
n2	0.55	0.82
n3	0.57	0.78
